# Stakeholders' Perceptions of Medical Leadership in Indian Medical Schools: A Thematic Analysis

**DOI:** 10.7759/cureus.81375

**Published:** 2025-03-28

**Authors:** Guru Thangiah Arun, Gugapriya TS, Ilavenil K, Kamala E

**Affiliations:** 1 Radiology, College of Medicine, Qatar University, Doha, QAT; 2 Radiology, Hamad Medical Corporation, Doha, QAT; 3 Anatomy, All India Institute of Medical Sciences, Nagpur, Nagpur, IND; 4 Anatomy, Karpagam Faculty of Medical Sciences and Research, Coimbatore, IND; 5 Anatomy, Trichy SRM Medical College Hospital & Research Centre, Trichy, IND

**Keywords:** competency-based education, curriculum, indian medical school, leadership training, medical education, medical leadership, qualitative research, ripple model, stakeholders perspective, thematic analysis

## Abstract

Background and objective

In recent times, the need for structured leadership development programs to produce effective leadership in healthcare professions globally has been highlighted. Understanding how stakeholders conceptualize leadership is key to ensuring the effectiveness of leadership training programs. Stakeholders’ perceptions of what makes an effective leader will influence their expectations about the content of such programs. Training programs are more successful if there is end-user acceptance of the underlying rationale of the program. Developing leadership development programs that echo and reflect user perceptions is key to the programs' success. If trainees’ expectations about the program are not met, they are less likely to have positive attitudes toward the program. Thus, it is important to understand different stakeholders’ perceptions about leadership and the currently existing leadership program curriculum. Very few studies in the literature have focused on the perceptions of diverse groups of stakeholders in the healthcare context. Studies focusing on the perceptions of medical leadership and leadership training in Indian medical schools are scarce. This study aimed to address this gap by exploring the perceptions of different stakeholders on medical leadership, current leadership training curricula, and factors influencing leadership training.

Methods

This was a qualitative study involving different stakeholders with leadership experience. We selected 20 participants with leadership experience. Stakeholder groups included five medical students (year four undergraduates, interns, postgraduates), five clinician leaders who are also department heads, five medical scientists (lecturer/tutor, assistant, and associate professors), and five institute leaders (associate dean, assistant dean, vice-dean, and dean). The participants were members of the institute’s undergraduate curriculum committee at a rural Indian medical school. We collected the data through semi-structured interviews and analyzed it using Braun and Clarke’s six-phase thematic analysis.

Results

Stakeholders expect the leaders to be role models and sources of inspiration who can communicate their vision, manage disagreements, and make decisions effectively. Stakeholders considered leadership curriculum, learners, faculty, institutional administrators, time availability and context of the institutions as factors that influence leadership training.

Conclusions

Based on our understanding of participants' perceptions, we highlight the need for a Specific, Measurable, Attainable, Relevant, Timely (SMART), and sustainable leadership curriculum. We propose the ripple model of leadership which could influence stakeholders’ identity, perceptions, competency, and actions that in turn would enhance enablers and reduce barriers to leadership training at all levels of the healthcare system to provide the desired outcome: effective healthcare leadership.

## Introduction

Leadership is a complex, multidimensional concept. The definition of leadership has evolved over time, from power over people to collaborating with people to achieve a specific goal [1]. Leadership is a process that is unique to a given context, where an individual who has followers influences the subordinates to act willingly to achieve a common goal [2]. The understanding and interpretation of leadership have a direct influence on the individuals’ capability and groups’ performance and, in turn, the organization’s effectiveness. This has led leadership development to be seen as a critical and important strategic focus for organizations [3]. Leadership is identified as essential in healthcare organizations too. Globally, healthcare systems expect doctors to manage clinical and organizational demands [4].

Medical leadership, which bridges the managerial and medical worlds, is increasingly important due to its recognized role in improving the quality of care, patient safety, cost-efficient care, and overall organizational performance [5]. Competent medical leaders are essential across all healthcare professions to meet the growing demands in healthcare to deliver standard education, research, and clinical practice. Healthcare professionals especially medical doctors - whether junior or senior doctors, both in and outside the hospital - are expected to showcase various aspects of leadership - make decisions in a tough situation, adapt to the situation, and respond quickly to demanding scenarios; they should have the ability to communicate effectively to bring out the best abilities from team members, maintain their right emotional quotient, and have the skills to get the job done.

These highlight the critical need for leadership training in healthcare professionals. For a newly qualified medical doctor to display various facets of leadership needs, it is crucial to start leadership training earlier and follow up throughout their careers [6]. Consequently, leadership training and development are considered essential in health professional curricula to influence people, provide direction, and achieve the intended goals of healthcare [7]. Despite the recognized importance of leadership, in the medical field, there exists a significant leadership gap due to a lack of structured leadership training programs, insufficient time to accommodate leadership training curricula in already tight medical curricula, disregard of medical leadership as a separate specialty, and lack of clear career pathway in medical leadership [8].

Global efforts are underway to develop competent leaders in healthcare by bridging the leadership gap. Many developed countries, including the UK, the USA, Canada, and Australia, have designed specific leadership frameworks, developed models, and are presently conducting leadership training programs for medical professionals in various stages of their careers [9]. These leadership training programs are designed to provide medical professionals with the necessary leadership skills to address the needs of expanding and evolving healthcare systems. Unlike in developed countries, the Indian medical curriculum lacks structured leadership training. The Public Health Foundation of India (PHFI) conducted an Innovation Collaborative study in 2015 that revealed the deficiencies in the present medical education curriculum for formal training in medical leadership skills [10].

Despite the growing requirement for leadership training for healthcare professionals and the inception of the Competency-Based Medical Education (CBME) curriculum in Indian medical schools [11], to date, in contrast to the developed countries, there is no structured framework of medical leadership training in Indian medical schools. The effectiveness of medical leadership frameworks and models designed in developed countries, such as the Medical Leadership Competency Framework (MLCF), CanMEDS Physician Competency Framework, NHS Health Care Leadership Model, and Dutch framework, is questionable in Indian medical schools. It is because those frameworks are all based on a competency framework and built on the concept of transformative/collective leadership. On the contrary, traditionally, medical leadership in India has been hierarchical.

Another reason those frameworks may not work in the Indian scenario is the vast differences in the cultural-social-political-economic context. This was dealt with by Jogulu and Wood [12] in 2008, who highlighted the significance of culture by stating that the assumption that Western theories and models will enhance the understanding of leadership in organizations in general may be erroneous in other cultures. This is because culture usually refers to unique lifestyles, traditions, and values shared by groups of people or societies. Each culture shaped by distinctive history, geography, religion, language, and social norms has its own unique way of interpreting the world, which in turn influences the perceptions, communications, and behavior of the people. Similarly, culture exerts influence on different views and expectations of what constitutes effective leadership in each society. This explains the divergence in attributes between leaders from diverse cultures that constitute leadership effectiveness. Due to this hierarchical nature of medical leadership in India and the inherent cultural context differences, the authors of this study assume that the medical leadership frameworks from developed countries may not have a successful outcome in Indian medical schools.

To ensure that the existing CBME curriculum in Indian medical schools has a successful outcome - to produce competent medical leaders - it is essential to understand the perceptions of various stakeholders on leadership and leadership training using the lens of leadership theories and approaches. Leadership theories, which have evolved over time, are frameworks and models that provide insight into how individuals with certain traits, behaviors, and styles make effective leaders and can lead and influence others effectively. Some common leadership theories include transformational leadership, situational leadership, servant leadership, charismatic leadership, distributed leadership, and transactional leadership. Each theory offers a unique perspective on leadership.

Transformational leadership is often associated with inspiring and motivating followers to achieve exceptional results. Situational leadership emphasizes adapting one's leadership style to fit the specific needs of the situation and the followers involved. Servant leadership emphasizes leaders serving the needs of their team members rather than focusing solely on personal power or success. Authentic leadership centers on being true to oneself, maintaining transparency, and building trust with followers. Transactional leadership is a leadership style that utilizes rewards and punishments to motivate and direct followers. Charismatic leadership is characterized by a leader who uses his or her communication skills, persuasiveness, and charm to influence others. Distributive leadership implies a model of shared, collective, and extended leadership practice where the emphasis is on interdependent interactions rather than individual and independent actions. Contingency theory suggests that effective leadership depends on the situation and the leader's ability to adapt. The three contemporary leadership theories that are shown to have the potential to inform leadership in different healthcare settings are transformational, authentic, and collective or shared leadership [1]. In this study, we aimed to understand the perceptions of stakeholders by using leadership theories relevant to healthcare settings.

A literature review on how stakeholders perceive leadership training programs revealed that many studies are conducted in the Western context or from a business perspective. Only a few of these studies focus on the perceptions of diverse groups of stakeholders in the healthcare context. According to NHS England (2022), the stakeholder in the context of medical education is anyone with a vested interest or stake in the decision-making and activities of designing an educational intervention [13]. The stakeholders in medical education are students, nurses, physicians, paramedics, physician assistants, healthcare practitioners, course designers, institutional/organizational administrators, university deans, state and national boards, patients, and patients' family members. The Stakeholder or Stockholder theory, which originated in the field of strategic management (Freeman, 1984) [14], states that an educational intervention should be holistic and involve the shared values of all stakeholders. Within medical education, this approach has been particularly influential in the field of curriculum creation and development, assessment strategies, and course construction [15]. Accordingly, we have included all the relevant stakeholders at the medical school level in this study, except the patients.

Literature reviews showed that previous studies focusing on the perceptions of medical leadership were from either developed countries or from urban sectors in developing countries. Though a few studies focusing on stakeholder perceptions of leadership are from the Indian context, none are from a rural Indian medical school context. Since perceptions and experiences change according to the context, we aimed to focus on the unexplored perceptions of individuals from the medical schools in the rural context. This study addresses a literature gap in studies exploring perceptions of diverse stakeholders on medical leadership and leadership training from Indian medical schools, specific to rural contexts. We aimed to explore the perceptions of different stakeholders on medical leadership, current leadership training curriculum, and factors influencing leadership training in existing undergraduate medical programs. Insights from this study will inform as to when, how, who, where, and what is expected in the leadership training program, assist medical educators and health administrators in evaluating existing leadership training programs, assess the needs and expectations of all stakeholders, revise current leadership curriculum, propose better sustainable leadership curriculum, create a supportive culture that encourages leadership development systemwide, and help plan further research exploring perceptions of patients as stakeholders and comparing perceptions of stakeholders from different contexts.

## Materials and methods

Study design and setting

This is an interpretative qualitative study using the constructivism paradigm. For perception exploration, qualitative research is the optimal method [16-17]. We conducted this study between January 7, 2023, and April 30, 2023, at Trichy SRM Medical College, located in the rural setting of Irungalur, Tiruchirappalli, India. Many of the participants were from Trichy SRM Medical College. At Trichy SRM Medical College, we also interviewed participants from two other medical colleges who were institute leaders/clinician leaders.

Participants

Study participants were medical students, faculty, and administrators in three medical colleges from rural backgrounds and involved in undergraduate medical programs. We classified them as stakeholder groups. Stakeholder groups included five medical students (year four undergraduates, interns, postgraduates), five medical scientists (lecturer/tutor, assistant, and associate professors), five clinician leaders who are also department heads, and five institute leaders (associate dean, assistant dean, vice-dean, and dean). The participants were members of the institute’s undergraduate curriculum committee.

Inclusion Criteria

Participants should be a stakeholder in undergraduate medical education. If the participant is a clinician leader/medical scientist/institute leader, they should have leadership experience and should be a member of the medical UG/PG curriculum committee. If the participant is a medical student, they should be a leader in one of the committees in the institute.

Exclusion Criteria

Medical students who were in their preclinical years and stakeholders involved in nursing/allied health science were excluded from the study. Though we agree with the fact that perceptions of preclinical students on leadership might provide unique insights, we excluded the preclinical students based on two reasons: (1) none of the preclinical students we screened were aware of the existing curriculum and (2) medical students were taking part in curriculum committee meetings as students' representatives only from year four onwards.

The research institute’s ethics committee approved the study - Reference No. 1333/TSRMMCH&RC/ME-1/2022-IEC No:214. We obtained permission only from the institute from which medical students and medical scientists participated. The permission from two other institutes was waived since the participants were institute leaders/clinician leaders who were in their respective institution’s ethics committees.

Data collection

We recruited the participants using purposive sampling. We initially screened 35 participants who showed interest in participating in the study. Of these, we selected 20 participants according to inclusion and exclusion criteria. We obtained written informed consent from the selected individuals. Twenty participants with experience in leadership positions and membership in the institute's UG curriculum committee volunteered to take part in the study. When selecting students for participation, we considered their experience in years as leaders in at least one of the institute's committees. We interviewed all participants face-to-face, and audio was recorded. We used the previously validated and tested interview questions from Bharwani et al. [18] and Chavan et al.'s [19] work after obtaining permission.

The interviews lasted between 45 minutes and one hour. None of the participants declined their consent to participate. We have elaborated on the participants’ responses to interview questions in the Results section. The questions focused on perceptions of medical leadership, existing leadership training, and factors perceived as enablers or barriers to the existing leadership training program for medical undergraduates. The responses to other interview questions addressed the ideal time for leadership training, topics that need to be included in the curriculum by the educational interventionist, teaching-learning and assessment methods that could be used by the educators in leadership training, and strategies for effective implementation of leadership curriculum.

To minimize the interviewer's bias, we followed the following strategies as the interviewer. a. Before interviewing, the interviewer learned about the interviewer’s biases and strategies to minimize them. b. We followed an interview protocol and used previously validated questions in the semi-structured interviews. c. We screened the individuals and selected the participants based on criteria to avoid and reduce 'impression bias' to the minimum. d. We practiced the interviews with three external researchers, obtained their feedback, and rectified them before the actual interview sessions. e. We used open-ended questions and asked the interviewees to explain their answers whenever necessary. f. We asked the same set of questions to all the participants. g. We verified the transcripts after the interview and addressed the queries and concerns.

Data analysis

The principal investigator (PI) of this study transcribed the audio-recorded interviews manually and became familiarized with the data. After the transcribed data were anonymized, we analyzed the data using Braun and Clarke’s six steps of thematic analysis [20]. The thematic analysis methodology provided a flexible framework for efficiently analyzing participant views and identifying patterns and themes in data [21]. We did not use any software for data analysis. We reviewed the transcripts and initially developed 10 thematic units. On re-examining the themes to verify whether the themes answered the research questions and refining the themes, we developed a total of 14 themes. We grouped the refined themes into four major themes and regrouped them into two categories to cover the focus of the research, and gave appropriate names that conveyed the collected information. We selected appropriate quotes from the transcribed data under the appropriate themes and linked them with the research findings [20]. Throughout this process, the PI engaged in a process of reflexivity to reflect on his own biases about leadership as a healthcare educator.

## Results

Twenty individuals with prior leadership experience and members of the medical curriculum committee participated in the study. The participants were divided into four groups, each with five people: administrators, clinical leaders, medical students, and scientists. Medical students (1-3 years), medical scientists (5-8 years), clinical leaders (10-19 years), and administrators (12-20 years) had leadership experiences. Three female and two male medical students were in the age range of 23-26 years. Four male and one female medical scientist were between 29-38 years of age. In each of the clinician leaders and administrators groups, three females and two males between 45-65 years of age participated (Table 1).

**Table 1:** Groupwise demographics of the participants

Stakeholder groups	Age group (years)	Leadership experience (years)
Medical students	23-26	1-3
Medical scientists	29-38	5-8
Clinician leaders	45-65	10-19
Administrators/institute leaders	45-65	12-20

**Figure 1:**
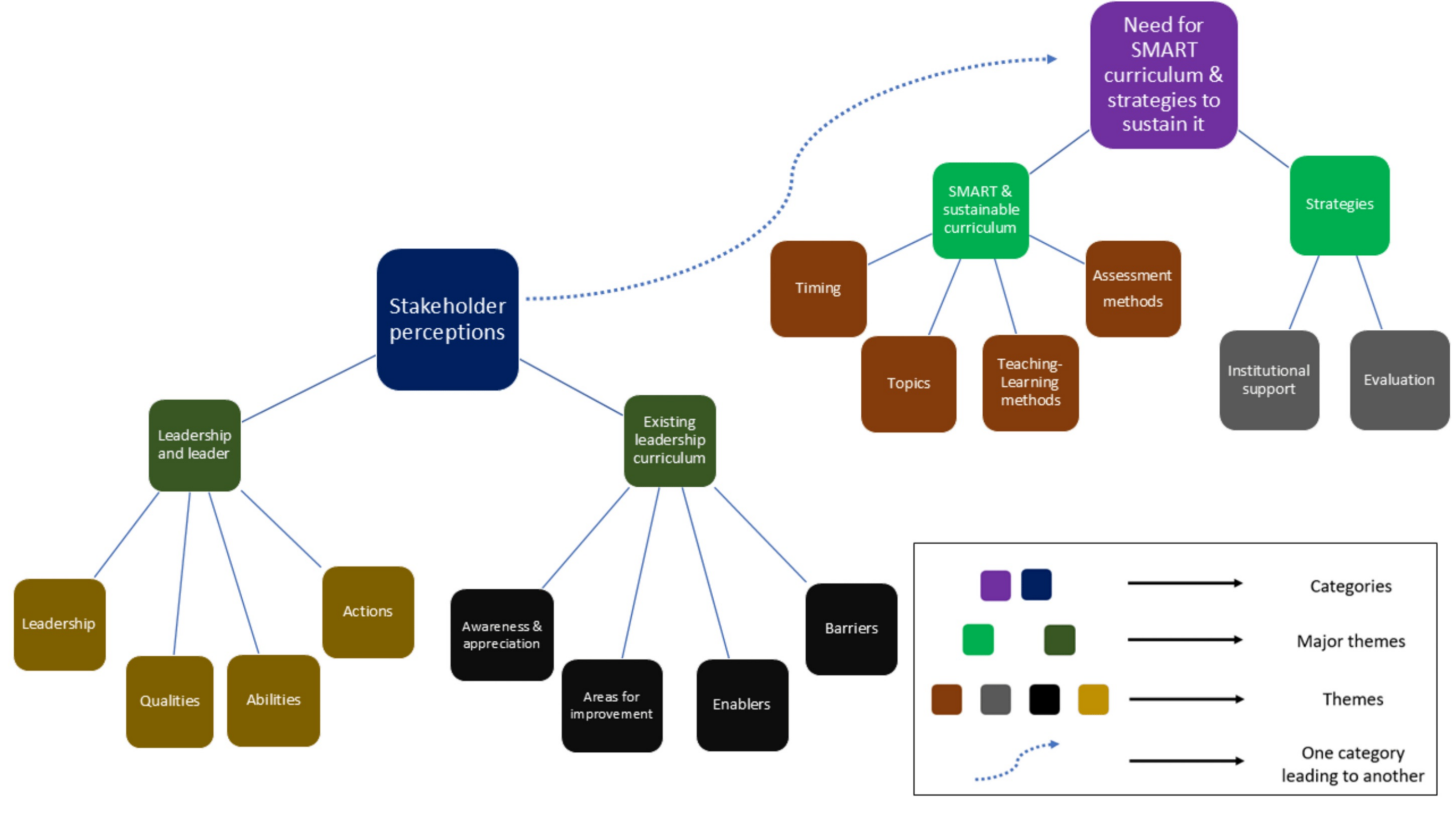
Thematic map The map shows the 14 themes, 4 major themes, and the overarching two categories; it also shows how stakeholders' perceptions lead to the need for recommended curriculum and strategies to sustain it (blue dotted curved line) Image credits: Guru Thangiah Arun

Fourteen themes emerged from the data and these were grouped into four major themes as follows: leadership and leader (leadership, qualities, abilities, and actions), existing leadership curriculum (awareness and appreciation, areas for improvement, enablers and barriers), SMART (Specific, Measurable, Achievable, Relevant, Time-based) and sustainable curriculum (focusing on timing, topics, teaching-learning methods, and assessment methods), and strategies (institution support and evaluation). We organized these four major themes under two overarching categories: stakeholder perceptions of leadership and the existing leadership curriculum, and the need for the SMART and sustainable curriculum and strategies to sustain it. Figure 1 depicts the thematic map and the interconnection between the categories.

Leadership and leaders

Defining Leadership

Most of the stakeholders indicated that leadership is about the activities of a leader while the leader is the person with certain qualities and skills who does the activities by behaving in a certain expected manner.

I think leadership is about the techniques and the methods used by a leader while leading the team to achieve a vision (clinician leader 3).

Regarding the meaning of leadership, most of the participants from all four groups perceived that leadership is about achieving a goal and not about position or hierarchy. There were minor differences in emphasis between student groups from the rest of the groups. While the former group focused on getting the job done, the rest of the groups, from their experiences, emphasized influencing team members to work together and achieve the common goal envisioned. 

Leadership is basically to get the job done, whatever it may be, in whichever discipline (medical student 2). 

Leadership is something where we must lead a team, take the initiative so that the people or the audience we lead progress, bring change, or achieve a vision (administrator 4).

Leadership Qualities, Skills, and Perceived Actions

Student group members considered leaders to be approachable, available, open to discussion, responsible, maintain integrity, and respectful to team members. Non-student groups mentioned two characteristics commonly: leader being an inspiration and a positive role model.

A leader should be an expert in his field so that he can inspire people; he should be a role-model by being trustworthy and emotionally intelligent in managing self and team-members (administrator 1).

Other qualities that were mentioned by the four groups were as follows: accountable, path setter, flexible/open, compassionate, empathetic, ready to lead, emotionally intelligent, trustworthy, reflective, self-motivated, and ethical.

All stakeholder group members consistently agreed upon the need for leaders to have good communication skills, conflict management, and decision-making skills. Members of clinician leader and medical scientist groups mentioned time-management, self-management, and people-management as essential for a leader. Administrator group members perceived technical competence, ability to collaborate, think strategically, manage teams effectively, organizational knowledge, delegation, and implementation skills as required skills of a leader.

A leader must have good communication skills and should actively listen. He/She must be a person who has a grasp of handling conflicts and taking decisions appropriately (medical student 3).

There was no common perception among the groups regarding the behaviors considered as actions of a leader. Student group members mentioned multi-tasking, talent recognition, and nurturing talent among team members as the actions of a leader. Medical scientists and clinical leader groups considered the following as the actions of a leader: appreciating and rewarding the contribution of team members, working together and motivating team members, not forcing subordinates, being diplomatic, and exhibiting confidence in their actions. Administrators differed from other groups’ perceptions and emphasized having a vision and being able to communicate it, engaging team members, being proactive to handle situations, and consensus-building during conflicts.

Leaders should have a vision and ability to communicate it clearly; I think a leader would delegate the work to the correct people by understanding their passion and motivating them to work together to promote change. He should be proactive at times of struggles and build consensus whenever there is a conflict among them (administrator 3).

Existing leadership curriculum

All the participants acknowledged their awareness of the recent leadership curriculum and its relevance in leadership development. They also expressed its drawbacks and the need for its revision. Regarding the enablers and barriers to leadership training, participants mentioned the following factors: students, faculty, institution, existing curriculum, time, and the context.

Awareness and Appreciation

All groups unanimously accepted the recently introduced CBME curriculum and recognized that a curriculum focusing on the role of a leader is necessary for learners. Every participant mentioned there was no such curriculum previously and appreciated its intention to develop leaders. However, except for the students’ group, the majority of the other group members emphasized that the existing curriculum has a lot of drawbacks and hence needs revision before proper implementation as follows: *“if refined/if done properly/if correctly implemented”.*

Previously there was no such part in the medical curriculum focusing on leadership. The new curriculum needs to be welcomed and given time. If its drawbacks get revised this curriculum will provide learners with ample opportunities and experiences for the learners to develop gradually into a leader (administrator 2).

Areas for Improvement

The commonly mentioned drawbacks by medical students and medical scientists were no explicit competencies given for the leader role, no structured teaching and assessment enrolled in the curriculum. Other drawbacks mentioned by the administrators and clinician leaders included the curriculum being very abstract, no clear-cut topics being given, failure to include interprofessional team activities, and failure to specify which department will deal with this leadership curriculum.

The existing leadership curriculum is given as a hidden curriculum. That's what I think is the problem. It doesn’t specify whether one or all departments are responsible. There is no specific time frame mentioned, no specific sessions given…. The assessment part is completely lacking. Because of that this is neither taught nor assessed (clinician leader 1).

Influencing Factors (Enablers and Barriers)

**Figure 2:**
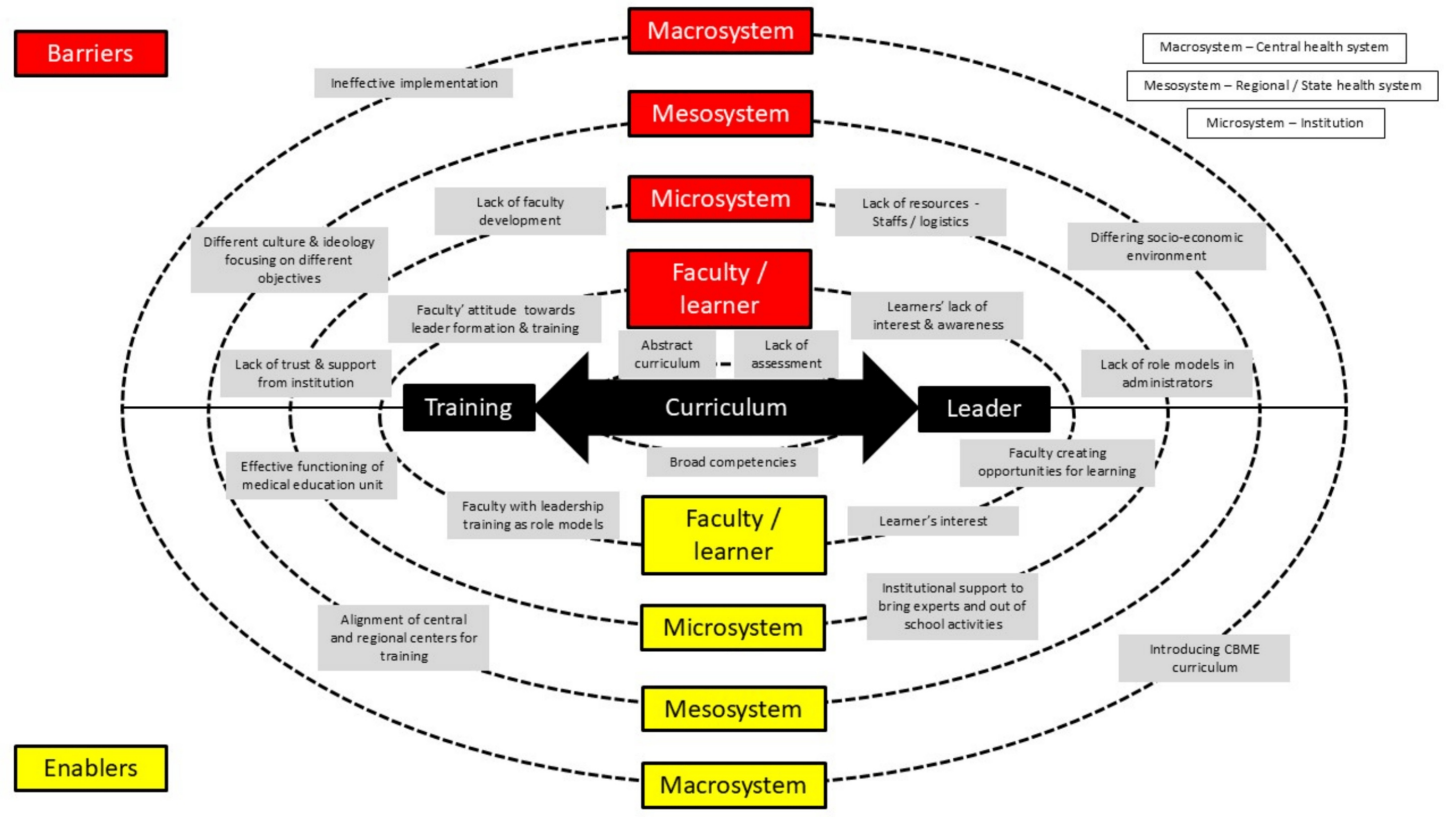
Enablers and barriers in leadership training programs at various levels of healthcare education system Image credits: Guru Thangiah Arun

There were divergences in the responses from all the participants regarding the factors they perceive as enablers and barriers for the leadership training in medical students. Members from all groups had contrasting perceptions, considering faculty/students/institution/context/time/curriculum/management and administrators as enablers or barriers. Figure 2 shows the enablers and barriers in leadership training programs at different levels in the healthcare education system.

The faculty perceived the following reasons as prime barriers: the faculty’s attitude towards preference for traditional curriculum, faculty giving importance to clinician role compared to leader, and their lack of training in CBME and leadership. On the other hand, the drawbacks of the existing leadership curriculum and ignorance of the new medical students entering undergraduate were reasons quoted as barriers. Both students and other stakeholder group members mentioned a lack of time. On the contrary, a few members from clinician leaders and medical scientists mentioned time as the overhyped variable and added that if the curriculum was properly rolled out, leadership could be taught adequately over the course of the undergraduate program. While the clinician leaders and administrators perceived the context of the institution as enabler, medical scientists quoted it as a barrier. Twelve out of fifteen non-student group members mentioned institution management as an enabler.

Context and the faculty in it play a major role. Both might act as an enabler or barrier. Faculty and administrators must understand the influence or the impact of context in the education system. A tertiary care hospital attached to a teaching institute might not be the same as a private institute in a rural setup. The students coming from different contexts differ widely in their level of exposure. So according to learners and according to the context, faculty must adapt and adopt facilitation and assessment techniques (clinician leader 2).

The only barrier I could see is the faculty who will not be very much into it. Because they don’t have training in leadership, no similar program previously in medical curriculum, and they don’t have the clear-cut idea. A handful of faculties may have the notion that leaders are born, so the mindset of the faculty needs to be changed (administrator 3).

## Discussion

The purpose of this research was to investigate stakeholders' perspectives on medical leadership and the elements that influence medical leadership training in existing undergraduate programs. Participants expressed the view that the leaders are made, and that individuals can be trained to demonstrate leadership abilities. This opinion is supported by the behavioral theory, which argues that leaders are made, not born, and more focus should be on leader’s actions rather than their personality traits [22-23].

It is crucial for all groups of stakeholders in an organization to have a clear and shared understanding of what leadership means and how its principles can be applied in leadership training and practices [24-25]. This study found that there were differences in the perception of leadership between the groups. Though our participants’ views on leadership differed, their views regarding the expected attributes, talents, and behaviors of a medical leader are like findings in a study examining leadership perspectives in Canadian medical schools [18] and cross-cultural leadership perspectives [26]. This similarity in outcomes to the literature about what is required of a leader can aid in the design of a fundamental medical leadership program that can be accepted over a broad horizon.

Understanding stakeholders’ expectations is critical when designing and developing leadership programs [27-29]; it will assist us in delivering content that is relevant to all stakeholders and meets their expectations. The results of this study revealed that stakeholder groups had distinct expectations regarding leadership and leaders’ attributes, which were different from those stated in their respective studies by Bharwani et al., Keluth et al., Gentry et al., Blumenthal et al., and Quince et al. [18,19,26]. For example, integrity and sense of passion were viewed as principal characteristics of a leader in Bharwani’s study while in this study inspiration and role-modelling were most viewed as leaders’ characteristics; technical competence and communication skills were endorsed as important for a leader in Bharwani’s study on contrary to communication skills, managing conflicts and decision making endorsed as important in this study. This difference in expectations among stakeholders in this study and Bharwani’s study can be explained by theories of expectancy [30-31] and contingency theories [32-34].

Theories of expectancy state that a person's choice of a certain action reflects their belief that such action will result in a desired outcome. The contingency theories explain how the actions of effective leaders are dependent on the context, environment, or situation of the surroundings. These leadership theories offer a unique perspective on leadership and can be applied in various contexts - such as healthcare, business, politics, private, public, and non-profit organizations - to guide individuals in developing leadership skills, leaders in making informed decisions and achieving organizational goals. Especially in India, with its multifaceted challenges in healthcare - large population, cultural and geographical variations across states, urban and rural settings, diversity in healthcare provision, limited resources, rising healthcare costs and increasing population demand, and its predominantly skewed distribution of doctors throughout the nation - understanding the evolution of leadership theories in healthcare sector is crucial for designing and providing an effective leadership training programs nationwide.

While leadership training focuses on the learners, this study highlights how other stakeholders and factors influencing leadership training must also be addressed. Participants cited learners’ lack of awareness about leadership importance as a barrier to leadership training. The faculty’s knowledge and skills gap in leadership and CBME curriculum and their preference for competencies focusing clinician over the leadership role were viewed as a few individual factors that could impede effective leadership training. Based on the responses of our participants, this study advocates faculty development programs that focus on learning objectives based on needs assessment [35].

A meta-analysis by Blume et al. [36] reports the importance of paying attention to context in leadership. Our participants said that a lack of logistics and human resources in the learning environment might cause this program to fail. They said that adequate skilled faculty and logistics are required for successful training. Their observation resonates with the best practices for leadership development [37]. In this study, the participants understood the role of institutional administrators, as well as the culture that prevails within it, as critical for the existing leadership curriculum to function. Other research, such as that by Avolio and Gardner [38], has also shown that organizational culture is a critical element for leadership to flourish.

Participants in this study supported the inclusion of leadership learning in the new CBME curriculum. This is a view that is also shared by Quince et al. [39] and Chavan and Bendriss [19]. Stakeholders said that the current curriculum is vague; it is irrelevant to learners and lacks an assessment component. The participants recommended a curriculum that clearly states what is expected of them as developing leaders and the standards to which the leaders must perform. This is consistent with the concept that setting explicit goals promotes performance [40].

**Figure 3:**
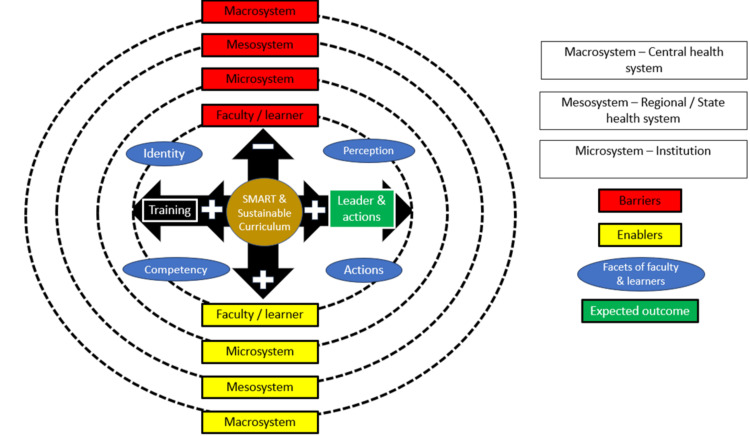
The ripple model - wherein the recommended SMART and sustainable curriculum causing ripple throughout the healthcare education system as depicted Image credits: Guru Thangiah Arun

By relating the emerged themes and categories, we have proposed a ripple model for leadership training (Figure 3) [41]. This model interconnects the themes and categories, and it focuses on system-wide leadership training by applying systems thinking [42]. In this model, the curriculum is believed to be the stone that could cause a ripple system-wide. It could influence all the stakeholders, reduce the barriers, and enhance the enabling factors for effective training mentioned by the participants. This model emphasizes the existing curriculum to be revised into a SMART and sustainable curriculum which is Specific in its objectives, where learning is made Measurable by including assessment, has Achievable competencies and goals according to the learner’s level, Relevant to the stakeholders’ needs and context, and has explicitly mentioned Time-bound training sessions with extended support for learning. This SMART curriculum should incorporate common elements of effective leadership development programs [8]. It must be based on stakeholder needs assessments; it should include relevant topics and be flexible to the context.

The ripple model recommends a sustainable curriculum that makes all faculty and learners explore their perceptions, values, and beliefs [43] and make them reflect on how their beliefs and actions impact local, regional, national, and global levels. Literature on Sustainable development states that sustainable development is necessary to meet the needs of the present without compromising the ability of future generations to meet their own needs [44]. A sustainable curriculum would be transformative rather than transmissive of knowledge [45-46]. A sustainable curriculum is critical to develop transformational leadership which is considered to have the potential to meet the demands of dynamic and complex healthcare settings. It engages the learners as co-creators in curriculum development instead of mere liaisons. A sustainable curriculum that actively engages the stakeholders in the change-making process will motivate them to periodically revise the curriculum alongside the institution according to the needs of the healthcare system [47]. A sustainable curriculum is based on seven principles: transformation and change, lifelong learning, systems thinking, envisioning a better future (problem-solving), critical thinking and reflection, participation, and partnerships for change [45-46]. Its principled foundation could make the stakeholders reflect on their identity and perception which in turn gradually has its impact on their competency and behavior.

Leaders who emerge from such SMART and sustainable curricular training with the desired attributes, competencies, and behaviors will have an impact on the leadership training retrospectively, and vice versa. The actions of the emerging leaders would have a ripple effect in institutions, communities, and national healthcare systems over time by reducing the barriers and enhancing the enablers of leadership training. Institutional administrators must scaffold and inspire the teachers and learners by assuring high-level commitment and support to deliver the curriculum's resources and logistics while creating a supportive culture in the setting. We believe, with the SMART and sustainable curriculum, influence from emerging leaders, and commitment from all stakeholders, the much-needed successful healthcare leaders can be developed. However, just like any other curriculum, this proposed curriculum also needs to be evaluated periodically as required by the educators focusing on whether it is aligning with the leadership program’s mission and vision, supporting differentiated instruction, and assessing evaluation methods. By doing so, educators can make informed decisions, refine, and ensure that the proposed curriculum reflects their values, addresses the diverse needs of their students, fosters a purposeful and inclusive educational experience for all learners, and provides effective means to measure student learning.

Limitations and implications of the study

This study has a few limitations, such as its small sample size and the fact that participants are from selected small groups; moreover, this was a single-center study in a rural context. Perceptions depend on the experiences of individuals and the context in which they prevail. The perception of an individual from a rural, resource-limited, public sector will vary from the urban, resource-rich, private sector. Similarly, the perceptions of an individual, group, or society are affected by the culture that exists locally. Hence, the findings from a single-center study will vary across different contexts. This study has not included the patients who are the consumers at the user-end of training. Qualitative data analysis and the approach to analysis are also subject to researcher bias.

Insights from this study will assist healthcare researchers, leaders, and program administrators to concentrate on the nontechnical skills and characteristics that are essential for successful healthcare leadership. This study demonstrated the viewpoint that leadership training that addresses the needs and expectations of all stakeholders is appreciated and valued by all involved stakeholders. Further studies can be done in rural areas and other contexts to compare with this study’s findings. This study could be extended to explore the perspectives of patients. This study will assist medical educators to evaluate and revise the current leadership training and development initiatives in Indian undergraduate medical education. The proposed ripple model of leadership training with special emphasis on the SMART and sustainable curricula should inspire educators and researchers globally to design leadership programs. By creating opportunities for faculty development and supporting faculty with the availability of resources for leadership training, institution administrators should commit to creating and maintaining a culture that supports and encourages leadership.

## Conclusions

The findings of this study underscore the importance of identifying exemplary faculty leaders and emphasize the need for context-specific leadership behaviors. This approach aims to cultivate future leaders capable of effectively articulating their vision, managing conflicts, and making decisions in complex, high-stress healthcare environments. Institutions should create a supportive culture, provide resources for training, ensure faculty development periodically, and encourage faculty who are interested in leadership roles. The suggested ripple model of leadership training could have a positive impact on all stakeholder groups at all levels of the healthcare system with its SMART and sustainable curriculum that emphasizes stakeholders' identity, perception, competency, and actions. It could also result in the crucially important desired outcome from CBME: effective leadership in healthcare.

## Appendices

Interview questions were received from the respective author of the below cited articles. I have permission from the author to use it in this study.

Bharwani A, Kline TJ, Patterson MB: Perceptions of effective leadership in a medical school context. Canadian Medical Education Journal. 2019, 10(3):e101 - e106. 10.36834/CMEJ.53370

Keluth Chavan A, Bendriss R: Leadership curriculum in medical education: exploring student and faculty perceptions in a US Medical School in Qatar. Journal of Healthcare Leadership. 2022, 14:163-73. 10.2147/JHL.S370645

Interview questions

Initial Questions

Whom do you look up to as a leader in your personal/professional life? What, in your opinion, makes that person a leader?

Leaders are born or leaders are made. Which one do you choose and why?

What do you think leadership is about?

What qualities do you expect in a leader?

What skills do you expect in a leader?

Intermediate Questions

What are your perspectives about existing leadership training for undergraduate medical students?

What knowledge and skills do you think are necessary for undergraduate medical students to become leaders in healthcare?

In what areas of your clinical curriculum did (medical students or you feel you) learn about leadership the most?

When do you think is the ideal time for leadership training in medical students? Why?

What factors do you perceive as barriers to effective leadership training in your college?

What, in your opinion, supports the existing leadership training in your college?

Closing Questions

Is there anything else you would like to tell me that you think might be useful?

If you are a curriculum director, what curricular change would you bring in existing leadership training in your college? What advice, strategies, and approaches would you suggest to support leadership development?

What leadership topics and skills do you think need to be focused on in leadership training?

In your opinion, how, when, and where should leadership development happen in your college?
